# Worshiping false Gods

**DOI:** 10.4103/0970-1591.32054

**Published:** 2007

**Authors:** Nitin S. Kekre

**Affiliations:** Editor in Chief, IJU, Professor, Department of Urology, Christian Medical College, Vellore - 632 004, Tamil Nadu, India. E-mail: editor@indianjurol.com

Evidence-based or Experience-based medicine? We surgeons are a peculiar lot as we often embrace newer treatments, newer technologies and operations with great enthusiasm without actually worrying about the quality of evidence to support the shift in our management plan. For example, there was no randomized trial between TURPs and open prostatectomy or PCNL versus open stone surgery. Today both these procedures have established a place beyond doubt and retrospective studies have unequivocally provided support to this approach. But does this approach always prove to be correct? The history of medicine is replete with many more procedures or treatments which entered with a bang but disappeared over a period of time as they did not stand the test of time. It has been well documented that the prescription of a particular drug goes up following a marketing seminar of a drug company. Pharmaceutical companies provide selective evidence which will help them to sell their product and it may not be of a good quality. I am sure Urologists haven't forgotten the lessons learnt from the famous “maximum androgen blockade” fiasco. And it is in this context that all of us need to be familiar with the basics of good quality evidence-based medicine. We must learn to look for quality evidence Dr. Dorairajan and colleagues review in this issue evidence-based medicine with special reference to urology with very practical examples. It is prudent that significant changes in the health policy must be based on good quality evidence. It can't be stated more succinctly than as stated by Murphy “*If we can never know the answer let us be honest and admit that we cannot. If we cannot produce proof, let us withhold the final stamp of intellectual approval until we do. It is better to be an agnostic forever than to worship false Gods”.*

Urinary incontinence is a socially distressing problem. The International Continence Society defines urinary incontinence as a condition of involuntary urine loss that is objectively demonstrable and is a social or hygienic problem. Urinary continence involves a complex interplay between micturitional physiology and functional ability. Urinary incontinence affects 17 million men and women in the US of which 85% are women. There is a general perception in India among the healthcare personnel that incontinence of urine is not a significant problem, even the medical curriculum does not pay enough attention to the diagnosis and management of urinary incontinence. There is no good quality data available on urinary incontinence either from our country or from the developing world. But there is no reason to believe that it would be any different to the developed world. The problem of urinary incontinence does not spare any religion, caste, race or nationality. People from the underdeveloped world may not be able to get proper medical advice. The governments of poorer countries have many important health problems to deal with - and are hardly expected to pay attention to urinary incontinence which was regarded till recently as symptoms only. Recently the WHO has awarded urinary incontinence a disease status and it is hoped that this would change the attitude of healthcare personnel as well as health planners towards this distressing condition. Since it is more common in females who are less empowered in our country, it leads to psychological morbidity including poor self-esteem, social withdrawal, depression, sexual dysfunction from embarrassment and curtailed social and recreational activities. Most of them suffer silently and do not seek medical help because of embarrassment. Many of them are not even aware that incontinence is treatable. Hence it is important that urologists and the Urological Society of India take a lead in the education of healthcare personnel, increase public awareness and work towards making the treatment of this condition affordable. With this in mind I had requested Dr. Gopal Badlani, internationally renowned urologist with special interest in female urology and incontinence to guest edit a symposium on this very important health problem. I express my gratitude to Dr. Gopal Badlani and his team who have brought forward the symposium on such a vast and difficult topic covering most of the aspects of urinary incontinence.

Premature ejaculation is the most common sexual dysfunction in men younger more than 40 years. The condition is complex since it involves the desire of both sexual partners either of whom or both may be at fault. The last decade has seen a significant growth in understanding the science of sexual dysfunction. Dr. Chris G. McMahon from the Australian Centre for Sexual Health, Australia, has written an exhaustive review on this topic in the symposium. I am greatly indebted to Dr. McMahon for his effort.

I am very happy to welcome Dr. Deepak Dubey and Dr. Rajeev Kumar, Assistant Professors of Urology from SGPGI and AIIMS on the editorial team of the Indian Journal of Urology as Assistant Editors. I am hopeful that their active contribution will provide a newer dimension to the journal.

With best wishes.

